# Single Cell T Cell Receptor Sequencing: Techniques and Future Challenges

**DOI:** 10.3389/fimmu.2018.01638

**Published:** 2018-07-18

**Authors:** Marco De Simone, Grazisa Rossetti, Massimiliano Pagani

**Affiliations:** ^1^Istituto Nazionale Genetica Molecolare INGM ‘Romeo ed Enrica Invernizzi’, Milan, Italy; ^2^Department of Medical Biotechnology and Translational Medicine, Università degli Studi di Milano, Milan, Italy

**Keywords:** T cell receptor repertoire, RNA sequencing, single cell analysis, bioinformatics, immune system

## Abstract

The peculiarity of T cell is their ability to recognize an infinite range of self and foreign antigens. This ability is achieved during thymic development through a complex molecular mechanism based on somatic recombination that leads to the expression of a very heterogeneous population of surface antigen receptors, the T Cell Receptors (TCRs). TCRs are cell specific and represent a sort of “molecular tag” of T cells and have been widely studied to monitor the dynamics of T cells in terms of clonality and diversity in several contexts including lymphoid malignancies, infectious diseases, autoimmune diseases, and tumor immunology. In this review, we provide an overview of the strategies used to investigate the TCR repertoire from the pioneering techniques based on the V segments identification to the revolution introduced by Next-Generation Sequencing that allows for high-throughput sequencing of alpha and beta chains. Single cell based approaches brought the analysis to a higher level of complexity and now provide the opportunity to sequence paired alpha and beta chains. We also discuss novel approaches that through the integration of TCR tracking and mRNA single cell sequencing offer a valuable tool to associate antigen specificity to transcriptional dynamics and to understand the molecular mechanisms of T cell plasticity.

## Introduction

Human T cells develop in the thymus from progenitors originating in hematopoietic tissues. During their development, they acquire the ability to recognize foreign antigens and provide protection against many different pathogens. This functional flexibility is guaranteed by the expression of highly polymorphic surface receptors called T cell receptors (TCRs). TCR is composed of two different protein chains. The vast majority of human T cells express TCRs composed of α (alpha) and β (beta) chains while a small subset expresses TCR composed of γ (gamma) and δ (delta) chains. αβ T cells are key mediators of the adaptive immunity and recognize antigens presented in association to major histocompatibility complex (MHC) Class I and Class II proteins. γδ T cells instead are not MHC-restricted and are involved in “innate like” responses in tissues. αβ T cells represent more than 90% of the total T cell population and are more diversified compared to γδT; for this reason the vast majority of the studies to characterize TCR (1) are focused on αβ T cells.

**Figure 1:**
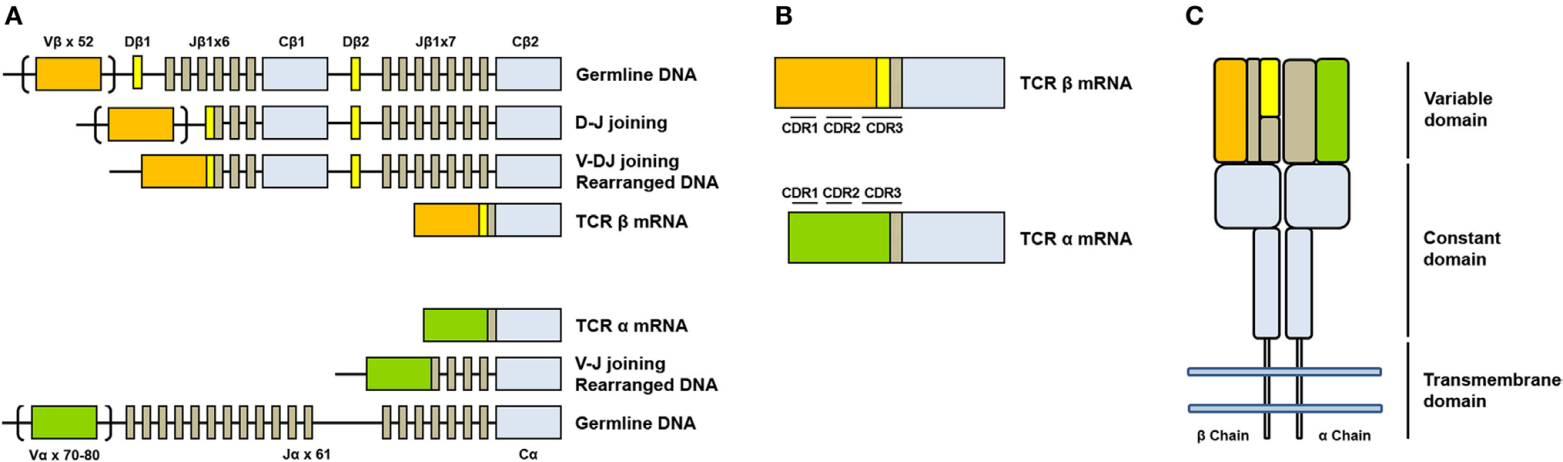
Somatic V(D)J arrangement in the alpha and beta chains. **(A)** Genomic organization and somatic recombination of *TCRB* and *TCRA* loci. Antigen repertoire diversity is guaranteed by a recombination step that progressively rearranges V, D, and J segments for T cell receptor (TCR) beta chains and V and J segments for TCR alpha chains. This variability (combinatorial diversity) is further increased by addition or deletion of nucleotides at the junction sites (junctional diversity). **(B)** Productive arrangements of beta and alpha transcripts. **(C)** Organization of TCR. TCR is composed by two subunits TCR alpha and TCR beta each organized in a constant region and a variable region responsible for antigen recognition.

The genes encoding alpha (*TCRA*) and beta (*TCRAB*) chains are composed of multiple non-contiguous gene segments which include variable (V), diversity (D), and joining (J) segments for *TCRB* gene and variable (V) and joining (J) for *TCRA* gene (2) (Figure 1A). The enormous diversity of T cell repertoires is generated by random combinations of germ line gene segments (combinatorial diversity) and by random addition or deletion at the junction site of the segments that have been joined (junctional diversity).

The sequence encoded by the V(D)J junction is called complementarity determining region 3 or CDR3. This sequence has the highest variability in both alpha and beta chains and determines the ability of a T cell to recognize an antigen peptide presented by the MHC molecule (3) (Figure 1B). The combinatorial variability is further increased by the subsequent heterodimeric paring of alpha and beta chains (Figure 1C) and total number of possible combination is estimated to exceed 10e18 (4). T cell repertoire is dynamic and directly reflects the diversity of immune responses: antigen presentation to a naïve T cell in fact, in association to co-stimulatory signals, drives a rapid clonal expansion of cells carrying identical TCRs to generate a population of “effector cells.” After antigen clearance, a reduced number of these cells remain in the blood as “memory cells.” The characterization of the TCR repertoire has always been of great scientific interest because it accurately describes T cell dynamics in a wide range of diseases, including malignancies (5, 6), autoimmune disorders (7), and infectious diseases (8, 9).

## TCR Analysis from Pioneering Techniques to Next Generation Sequencing

Pioneering experiments to dissect the T cell repertoire were performed at protein level using flow cytometry and a combination of monoclonal antibodies against the TCRBV subgroups. This approach is both qualitative and quantitative but limited by the availability of specific monoclonal antibodies and did not provide any information about CDR3 diversity (10). The first genomic based approaches, instead, were based on the analysis of CDR3 sequence length distribution in a population. This technique, called Immunoscope or CDR3 Spectratyping (11) is based on the electrophoretic analysis of PCR fragments derived from amplification of TCR transcripts across the CDR3 region using primers specific for the different variable segments and the constant region. Immunoscope compares the relative frequencies of different length products within an individual TCRBV subfamily, which assume a Gaussian distribution in the case of a polyclonal population while it is skewed in the case of clonal enrichment. The first molecular approaches used to interrogate the TCR repertoire at the nucleotide sequence level were based on traditional molecular cloning and Sanger sequencing (12, 13). This approach provided a more specific description of TCR repertoire but it was not powerful enough to estimate the huge TCR diversity. The real breakthrough in the characterization of the immune repertoire came from the introduction of highly sensitive high-throughput sequencing techniques for massive parallel sequencing of millions of DNA molecules instead of single clones to provide a comprehensive knowledge of the TCR arrangement (alpha chain, beta chain, or both) including V–J segments and the complete CDR3 sequence. The current sequencing techniques employ a target enrichment step starting from genomic DNA or cDNA to both increase the sensitivity and reduce the sequencing costs. The commonly used enrichment strategies include multiplex PCR, RNA baits enrichment, and 5′ RACE PCR. Multiplex PCR strategies employ a multiplex pool of forward PCR primers complementary to all the possible V segments and a pool of reverse primers designed on the J segments (if starting from genomic DNA) (8) or on the constant region of alpha and beta chain (if starting from cDNA) (14). Both approaches present advantages and drawbacks. PCR enrichment starting from cDNA has many advantages over genomic DNA: (a) it is less biased by PCR artifacts because the amplified fragments do not include introns and are, therefore, smaller; (b) cDNA analysis detects only productively arranged segments (the “expressed” alpha and beta chains); and (c) it enables an easier detection of the less represented sequences because mRNA transcripts are more abundant than their template genomic DNA. Bait based enrichment employs RNA baits to capture TCR sequences directly from DNA or RNA sequencing (RNA-seq) libraries which are re-amplified after capture. Baits are specific for the alpha and beta transcripts and are usually conjugated to magnetic beads. This procedure requires few amplification cycles reducing the potential of PCR related biases (15, 16). The third approach is a transcript based approach that employs a 5′RACE after a template-switch step. RNA is reverse transcribed by an enzyme endowed with terminal transferase activity that adds a stretch of non- template dCTPs at the 3′ end of the cDNA. A non-template-switch oligonucleotide containing a poly-G tract then binds to the non-template stretch and allows the reverse transcriptase to switch template and to go on extending the template up to the end of the oligonucleotide. The template-switch oligonucleotide contains a universal sequence shared by all the transcripts including TCR chains. A forward primer designed on this sequence is then used together with reverse primers designed on the constant region of the alpha and beta chains to enrich for TCR transcripts amplifying fragments that can then be processed to generate sequencing libraries (16, 17). The use of genomic DNA as starting material is instead more challenging because enrichment is usually performed by multiplex PCR but the presence of introns and amplification of longer fragments can introduce more technical biases. In addition, non-productive rearrangements are also amplified and sequenced complicating the analysis of expressed repertoire. This revolutionary approach was first used to characterize the TCR repertoire diversity in healthy individuals (18) and rapidly adapted to the repertoire analysis in different pathological settings like tumor immunology (5) and autoimmunity (19) and in several clinical applications like the monitoring of hematopoietic cell transplantation (20). Beta chain has always been the main target in all the TCR repertoire studies due to its higher diversity related to a larger combinatorial potential compared to alpha chain. Moreover, beta chain represents an “unique label” for a T cell: T cells undergo a process called “allelic exclusion” that leads to the generation of only one productively arranged beta chain gene, while both the alpha chain alleles can be expressed (21). The limitation of the “bulk” approach resides in the lack of information about the pairing of the alpha and beta chain, which really reflects the biological function of a T cell *in vivo* and can be achieved only with single cell analysis. Single cell approaches for TCR repertoire analysis employ two main strategies: a direct target amplification and sequencing starting from single cell cDNA or the TCR reconstruction from single cell RNA-seq data.

## Single Cell TCR Enrichment and Sequencing

**Figure 2:**
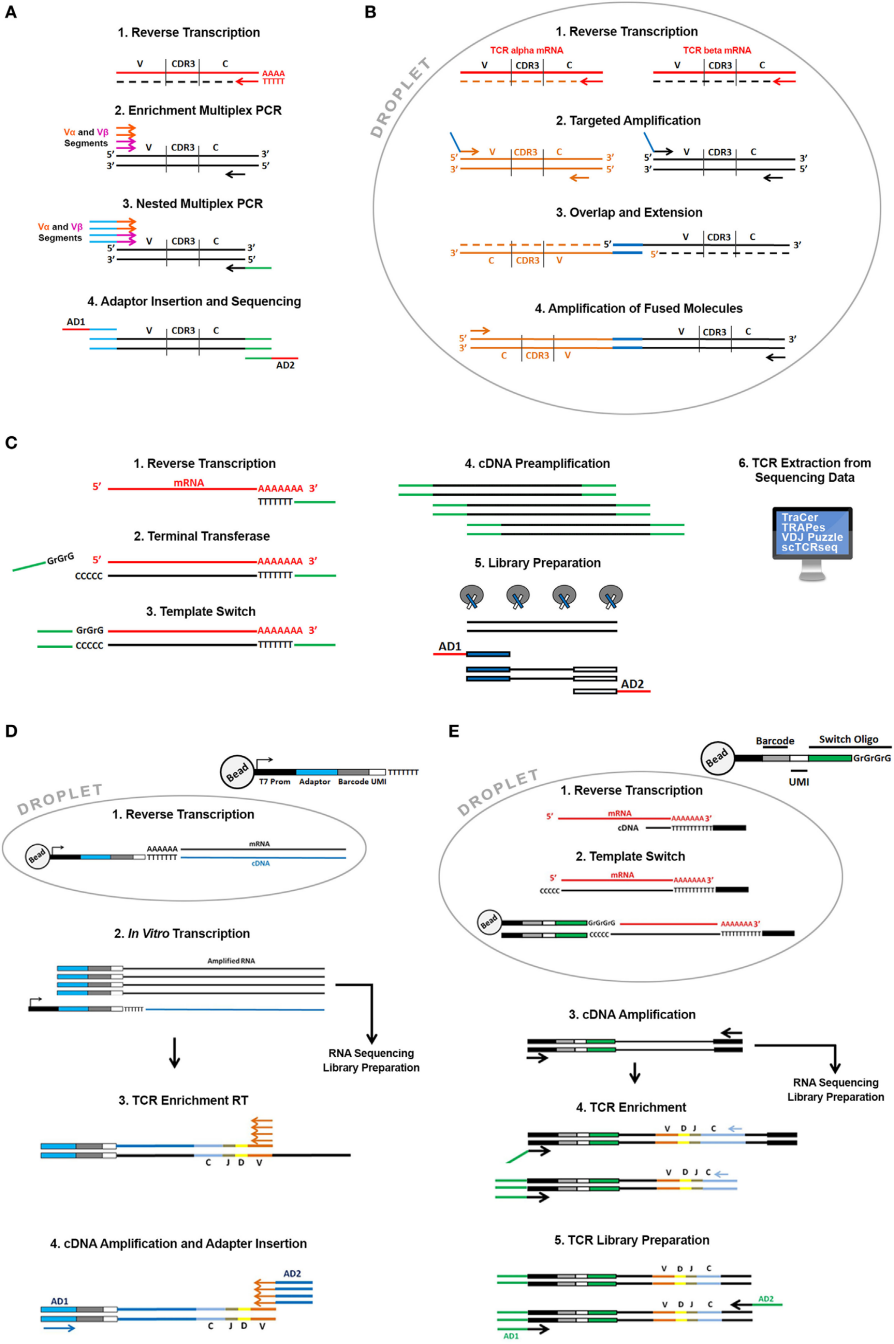
Overview of the available single cell T cell receptor (TCR) sequencing approaches. Direct TCR enrichment and sequencing. In **(A)** single cell TCR transcripts are enriched by a multiplex PCR performed after RT reaction using a pool of forward primers spanning all the annotated productive V alpha and V beta fragments and reverse primers designed on the constant region of alpha and beta chains. Barcoded adaptors then are added by PCR enabling pooling and sequencing by Next-Generation Sequencing. In **(B)** cells are captured in water-in-oil droplets using microfluidic emulsion-based devices along with specific RT and PCR reagents. In each droplet, TCR alpha and beta transcripts of a single cell are specifically reverse transcribed with RT primers designed on the constant region of alpha and beta chain. cDNA is successively amplified using a pool of forward primers designed on all the alpha and beta segments and reverse primers designed on the constant region. Alpha and beta primers contain overlapping sequences at their 5′ends that enable the synthesis of TCR alpha and beta fusion sequences through an overlap-extension mechanism. Fused molecules are pooled breaking the emulsion and further enriched by a nested amplification and sequenced. TCR reconstruction from single cell “full length” RNA sequencing (RNA-seq) data. **(C)** Single cells sorted in plates or captured using microfluidic devices are lysed, and total mRNA is reverse transcribed with an oligo dT primed reaction. Through a template-switch mechanism a universal sequence is added at the 5′ end of the transcript. This sequence, shared with the dT primer used in the RT reaction is then used to amplify cDNA before library preparation. In the library preparation step, full-length cDNA is “tagmented” using Transposase and Tag sequences inserted by transposase are then used to amplify cDNA and to insert barcoded sequencing adaptors (AD1 and AD2). Libraries are then sequenced and TCR sequences can be extracted from all transcriptome using dedicated bioinformatics algorithms (TraCer, TraPes, VDJ Puzzle). Pairing Single cell TCR sequencing and RNA-seq using emulsion-based protocols. **(D)** Thousands of cells in parallel are partitioned into oil-in-water droplets. After a lysis step their mRNA is reverse transcribed using a pool of specific RT primers containing the same “cell barcode” used to tag the cell transcriptome, a Unique Molecular Identifier (UMI). UMI is different for each primer enabling the digital counting of mRNA transcripts and sequencing of the T7 promoter. cDNA is then amplified by *in vitro* transcription. After amplification barcoded RNAs are pooled and processed together. Amplified RNA is then used as template to enrich for TCR sequences and to generate RNA-seq libraries according to the InDrop protocol. During RNA-seq library preparation RNA is fragmented and only the 3′ end of transcripts is sequenced. For TCR enrichment amplified RNA is reverse transcribed using a pool of RT primers spanning V alpha and V beta segments and then amplified using “internal” V alpha and beta primers and primers designed on the constant regions. During this PCR reaction sequencing adaptors are also added (AD1 and AD2). **(E)** Thousands of cells in parallel are partitioned into oil-in-water droplets. After a lysis step their mRNA is reverse transcribed using an oligo dT primer. Through a template-switch mechanism, a primer containing a cell barcode and a UMI is added at the 5′ end of the transcript. After RT reaction droplets are broken and cDNAs pooled and amplified using external primer designed on dT and switch oligonucleotides, respectively. Amplified full-length cDNA is then used as template to enrich TCR sequencing and and/or fragmented and processed to generate RNA-seq libraries. TCR enrichment is performed using nested PCR with a forward primer spanning the switch oligo and reverse primers designed on the constant region of alpha and beta chains. PCR products are then partially fragmented and sequencing adaptors are added (AD1 and AD2).

The first attempts to sequence single cell TCR alpha and beta chains used multiplex PCR strategies associated to Sanger sequencing (22) or high-throughput sequencing (23). Hans and colleagues characterized the heterogeneity of T lymphocytes infiltrating human colorectal carcinoma using a combined multiplex PCR approach to enrich both TCR sequences (Figure 2A) and a pool of “phenotyping genes” from the same cells. They also implemented a PCR-based single cell barcoding strategy to pool all the amplicons and sequence them by NGS. A barcode is a short nucleotide sequence that uniquely tags cell transcripts and is used to trace mRNA transcripts back to their cellular origins. Through this original approach they could associate the TCR sequence to specific T cell subsets with different functions. A similar single cell barcoding strategy was used to set up high-throughput methodologies to identify clones sharing the same alpha and beta TCR sequences and applied to tumor-infiltrating lymphocytes from breast and lung cancer (24).

A real breakthrough came with emulsion-based PCR techniques (Figure 2B). These techniques employ devices that pump oil-in-water emulsion where thousands of single cells can be captured into droplets that work like tiny reaction chambers along with primer and PCR reagents. This approach allows to drastically increase the number of cells processed in parallel and enables the generation of representative single cell alpha and beta TCR gene libraries fused together. In detail, alpha and beta TCR mRNAs are released in each single droplet after cell lysis and are reverse transcribed and amplified using multiplex PCR employing primers with overlapping ends. In a subsequent reaction the overlapping ends anneal allowing the 3′ end of each strand to prime the 3′ extension of the complementary end (25). This step generates a fusion fragment containing both the alpha and the beta sequence; fusion fragments are then further amplified in a PCR containing “blocking” primers that prevent unfused fragments to be amplified (26). Turchaninova and colleagues applied this technology to describe the T cell repertoire from human blood but this approach has been extensively applied in many areas of research including cancer (27) and recently readapted in an ultra-highthroughput platform that allows the TCR paired sequencing of millions of cells (28).

## TCR Reconstruction from Single Cell RNA-seq Data

The development of single cell RNA-seq technologies opened also new perspectives for TCR analysis. To date, many different protocols have been developed that mainly differ in the cell isolation methods, cDNA synthesis and amplification, and in the library preparation steps (29). Single cell isolation methods have rapidly evolved in the past few years from manual micromanipulation to high-throughput isolation using microfluidics or emulsion-based platforms and this has provided huge advantages not only in terms of throughput but also in terms of sensitivity and accuracy due to very small reaction volumes employed (30). All the sequencing protocols are characterized by a reverse transcription and an amplification step before library preparation. The commonly used protocols differ in the amplification step that can be performed by PCR or *in vitro* transcription and are divided into two groups: tag based or full-length cDNA sequencing protocols.

Tag-based strategies introduce a “cell barcode” during reverse transcription reaction providing the possibility to “tag” the whole cDNA pool of a single cell (31–33). “Tag strategies” have exponentially increased the throughput because tagged cDNAs can be pooled during the amplification and library preparation drastically reducing costs and experimental time. The main disadvantage is that these protocols lose the full-length transcript coverage following cDNAs fragmentation during library preparation and have lower sensitivity (lower number of detectable genes) compared to full-length strategies. Full-length strategies are instead more expensive and time consuming (cDNA pool from each cell is processed independently to generate a single sequencing library) but are more sensitive and can provide a broader range of information concerning isoforms, splicing events, and single nucleotide polymorphisms. Single cell RNA-seq data generated using full-length approaches have been exploited to extract information about T cell repertoire and heterogeneity. The assembly of full-length (TCR) sequences from scRNA-seq data allows for alpha and beta chain sequence pairing (not possible when performing “bulk” analysis) and for the integration of clonality information with the whole transcriptome of a single T cell. This approach also presents non trivial analysis issues: the canonical reference-based assembly methods that rely on the alignment of reads to a “reference” genome are in fact biased by the somatic rearrangements and mutations (CDR3 is specific for each TCR) and the lack of a complete “reference” genome has required the development of *de novo* assembly based bioinformatic tools. All the available tools basically combine reference-based assembly (reads are aligned to annotated gene segments) and *de novo* assembly (to reconstruct the CD3 region). One of the first tools developed to reconstruct paired TCR alpha and beta chains is called TraCer (34). To validate TraCeR perfomance, Stubbington and colleagues generated single cell RNA-seq data using the SMART-seq protocol (Figure 2C) and the FluidigmC1 System (Fluidigm Corporation) isolated from mice spleen. In order to validate the identified clonotypes, they used an experimental approach to enrich TCR sequences using a multiplex PCR-based approach starting from the same cDNA used for sequencing libraries generation that was sequenced in parallel by NGS. A good correlation among the two strategies confirmed the efficiency of the approach. In the same paper, they applied this analysis to a mouse model of *Salmonella* infection and were able to monitor the expanded clonotypes upon infection. The power of this approach was the association of TCR clonotypes to a specific gene expression profile that provided a detailed molecular description of the whole CD4+ T cell population and monitored the dynamics of CD4+ T cell subsets upon infection. This same approach was applied to dissect the heterogeneity of T cell subsets to address several biological questions in particular related to “tumor immunity.” The role of T cells in cancer has been extensively studied in the past few years and CD8+ T cells and CD4+ T regulatory cells have been broadly described to inhibit or promote tumor progression, respectively, in several types of cancer (35, 36). Recently, Zheng and colleagues used TraCer to dissect the landscape of T cell infiltrating liver cancer. They analyzed the clonal enrichment of infiltrating Treg and CD8+ T cells in order to understand the molecular mechanisms underlying lymphocyte recruitment in the tumor microenvironment. Through the dissection of the TCR repertoire they came to the conclusion that Treg cells do not present clonal enrichment in the tumor suggesting recruitment from the periphery while CD8+ T cells are clonally enriched suggesting clonal activation and expansion inside the tumor (37). Along the same lines of research, other tools were successively developed including scTCR Seq (38), TRAPes, applicable to short-reads single cell RNA-Seq libraries (39) and VDJ Puzzle that allows the simultaneous analysis of gene expression and TCR diversity and that was developed and validated on antigen-specific circulating CD8+ T cells (40).

## New Technologies and Perspectives

More recent attempts to modify tag-based strategies promise to couple RNA-seq and TCR sequencing from the same cells. These approaches have the great advantage to drastically increase the number of cells processed in parallel which is crucial to characterize very rare T cell populations.

A very recent publication investigated the T cell repertoire in mice and human Treg cells (41). In this paper, Zemmour and colleagues analyzed Treg cell transcriptional phenotypes using different single cell RNA-seq approaches. They showed that Treg cells can display a broad heterogeneity with some highly activated subpopulation which seems transcriptionally related to T conventional cells. They also showed that the Treg sharing the same TCR were more transcriptionally similar than Treg with different antigen specificity providing evidence that Treg plasticity can be strongly influenced by TCR shaping. To analyze the TCR repertoire, Zemmour and colleagues used a modification of the emulsion-based InDrop protocol (Figure 2D) that is currently used for 3′ prime end counting (33).

In detail, cells are captured into droplets through the formation of a water-in-oil emulsion through microfluidic devices. Cells are captured along with lysis buffer, reagents, and specifically barcoded primer that prime RT reaction. Barcoded cDNAs are synthesized from thousand of cell in parallel. After reverse transcription cDNAs from different cells are pooled by breaking the droplets, linearly amplified using *in vitro* transcription and then sequencing libraries are prepared. As already described in this review, this pooling strategy exponentially increases the throughput because thousands of cells can be pooled in a single library. The fragmentation step, though, sacrifices all the information derived from the 5′ end of the mRNA including the CDR3 region. Zemmour and colleagues overcame this problem by introducing a TCR enrichment step after linear amplification using a multiplex RT pool spanning all the V alpha and beta segments. This step generates TCR alpha and beta libraries sharing the same barcode with the whole transcriptome libraries (Figure 2D). Very recently 10x Genomics introduced a similar approach that couples alpha and beta chain sequencing to transcriptome analysis of thousands of single cells in parallel (Figure 2E). The approach employs a commercial emulsion-based microfluidic platform (Chromium 10x) that enables the generation of amplified cDNA used for both single cell RNA-seq libraries preparation and TCR target enrichment and sequencing. TCR enrichment is performed on amplified cDNA by PCR using reverse primers designed on the constant regions of alpha and beta chains and a universal forward primer designed on an oligonucleotide sequence added at the 5′ through a template-switch mechanism during the second strand synthesis. Each oligonucleotide contains a unique cell barcode to tag the whole transcriptome of a cell, including TCR transcripts.

## Conclusion

T cell receptor repertoire analysis has become a fundamental tool to understand the biology of T cells in healthy individuals and in several pathological conditions and is currently applied not only to study the biology of immune-mediated diseases but also to monitor the immune responses to therapies. Pioneering techniques such as CD3 spectratyping had been extensively exploited to provide information on clonal expansion but the real revolution in terms of throughput and applications was introduced by the development of NGS techniques. Sequencing the TCR of thousands of cells in parallel is a powerful instrument to dissect the complexity and diversity of the T cell response repertoire. The main limit of this technology relies in the impossibility to pair alpha and beta sequencing which impairs our understanding of what happens *in vivo*. This critical issue has been recently overcome with the fast development and expansion of single cell technologies that provide sequence information on paired alpha and beta chains of individual cells. A further level of complexity derives by the efforts to associate TCR repertoire and gene expression profile from the same cells. This kind of analysis provides an unbiased classification of a population of interest and the association of the transcriptional landscape of each cell with its TCR. This approach promises to open new avenues to describe the specific clonality of immune cell subsets even of the poorly characterized phenotypic subtypes and gives the opportunity to monitor the effect of transcriptional dynamics in strict association to clonality.

## Author Contributions

MDS, GR, and MP designed the article concept and scope and reviewed the article. MS wrote the manuscript and conceptualized the figures.

## Conflict of Interest Statement

The authors declare that the research was conducted in the absence of any commercial or financial relationships that could be construed as a potential conflict of interest.
